# Supplementary material for: Impact of health promotion strategies on HPV vaccination uptake: A descriptive epidemiological study (2019–2024)
Source: PLoS One. 2025 Sep 4;20(9):e0331592. doi: 10.1371/journal.pone.0331592 (PMC12410811; doi:10.1371/journal.pone.0331592)
Supplement: S1 Table — (DOCX) [file pone.0331592.s001.docx]

**S1 Table. Post hoc pairwise comparisons of the number of vaccinated boys by year of vaccination (2019–2024).**

| Year | Comparable years | M ^a^ | p ^b^ | 95% CI |
| --- | --- | --- | --- | --- |
| 2019 | 2020 | -.333 | 1.00 | -29.21 to 28.55 |
|  | 2021 | -1.583 | 1.00 | -30.46 to 27.30 |
|  | 2022 | -26.750 | .085 | -55.63 to 2.13 |
|  | 2023 | -40.833* | .001 | -69.71 to -11.95 |
|  | 2024 | -70.750* | < .001 | -99.63 to -41.87 |
| 2020 | 2021 | -1.250 | 1.00 | -30.12 to 27.63 |
|  | 2022 | -26.417 | .092 | -55.30 to 2.46 |
|  | 2023 | -40.500* | .001 | -69.38 to -11.62 |
|  | 2024 | -70.417* | .000 | -99.30 to -41.54 |
| 2021 | 2022 | -25.167 | .123 | -54.05 to 3.71 |
|  | 2023 | -39.205* | .002 | -68.13 to -10.37 |
|  | 2024 | -69.167* | < .001 | -98.05 to -40.29 |
| 2022 | 2023 | -14.083 | .708 | -42.96 to 14.80 |
|  | 2024 | -44.000* | < .001 | -72.88 to -15.12 |
| 2023 | 2024 | -29.917* | .038 | -58.80 to -1.04 |

^a^ Mean; ^b^ p value.
