# Supplementary material for: Impact of health promotion strategies on HPV vaccination uptake: A descriptive epidemiological study (2019–2024)
Source: PLoS One. 2025 Sep 4;20(9):e0331592. doi: 10.1371/journal.pone.0331592 (PMC12410811; doi:10.1371/journal.pone.0331592)
Supplement: S2 Table — (DOCX) [file pone.0331592.s002.docx]

**S2 Table. Post hoc pairwise comparisons of the number of vaccinated girls by year of vaccination (2019–2024).**

| Year | Comparable years | M ^a^ | p ^b^ | 95% CI |
| --- | --- | --- | --- | --- |
| 2019 | 2020 | -28.667 | .766 | -91.81 to 34.48 |
|  | 2021 | -47.083 | .257 | -110.23 to 16.06 |
|  | 2022 | -96.917* | < .001 | -160.06 to -33.77 |
|  | 2023 | -98.917* | < .001 | -161.56 to -35.27 |
|  | 2024 | -116.417* | < .001 | -179.56 to -53.27 |
| 2020 | 2021 | -18.417 | .955 | -81.56 to 44.73 |
|  | 2022 | -68.250* | .027 | -131.39 to -5.11 |
|  | 2023 | -69.750* | .022 | -132.89 to -6.61 |
|  | 2024 | -87.750* | .002 | -150.89 to -24.61 |
| 2021 | 2022 | -49.833 | .202 | -112.89 to 13.31 |
|  | 2023 | -51.333 | .176 | -114.48 to 11.81 |
|  | 2024 | -69.333* | .023 | -132.48 to -6.19 |
| 2022 | 2023 | -1.500 | 1.00 | -64.64 to 61.64 |
|  | 2024 | -19.500 | .944 | -82.64 to 43.64 |
| 2023 | 2024 | -18.000 | .960 | -81.14 to 45.14 |

^a^ Mean; ^b^ p value.
