# Supplementary material for: Impact of health promotion strategies on HPV vaccination uptake: A descriptive epidemiological study (2019–2024)
Source: PLoS One. 2025 Sep 4;20(9):e0331592. doi: 10.1371/journal.pone.0331592 (PMC12410811; doi:10.1371/journal.pone.0331592)
Supplement: Attachment — Submitted filename: Response to Reviewers.docx [file pone.0331592.s005.docx]

PONE-D-25-25832

Impact of Health Promotion Strategies on HPV Vaccination Uptake: A Descriptive Epidemiological Study (2019–2024)

PLOS ONE

**Editor’s comments**

Dear Dr. Štrbac,

Thank you for submitting your manuscript to PLOS ONE. After careful consideration, we feel that it has merit but does not fully meet PLOS ONE’s publication criteria as it currently stands. Therefore, we invite you to submit a revised version of the manuscript that addresses the points raised during the review process.

I would like you to go thoroughly across the reviews and check/change your paper in accordance with the suggestions reviewers made. I really think they made an effort to improve your findings/paper, and would like you to accept and implement everything they suggest. Please explain your changes in rebuttal letter in details. I find your paper very interesting and would like to see it published but some of the comments will certainly add specific value.

Please submit your revised manuscript by Aug 17 2025 11:59PM. If you will need more time than this to complete your revisions, please reply to this message or contact the journal office at [plosone@plos.org](mailto:plosone@plos.org). When you're ready to submit your revision, log on to <https://www.editorialmanager.com/pone/> and select the 'Submissions Needing Revision' folder to locate your manuscript file.

**Authors’ response:** Dear Iskra Alexandra Nola, thank you for forwarding the reviewers’ comments.

We have carefully considered all their suggestions and agree with the majority of them. We hope that our responses and the revisions made throughout the manuscript will be sufficient to support its acceptance. Our responses to the journal editorial board's requests and the reviewers’ suggestions are, as in this case, highlighted in yellow.

Please include the following items when submitting your revised manuscript:

- A rebuttal letter that responds to each point raised by the academic editor and reviewer(s). You should upload this letter as a separate file labeled 'Response to Reviewers'.
- A marked-up copy of your manuscript that highlights changes made to the original version. You should upload this as a separate file labeled 'Revised Manuscript with Track Changes'.
- An unmarked version of your revised paper without tracked changes. You should upload this as a separate file labeled 'Manuscript'.

If you would like to make changes to your financial disclosure, please include your updated statement in your cover letter. Guidelines for resubmitting your figure files are available below the reviewer comments at the end of this letter.

If applicable, we recommend that you deposit your laboratory protocols in protocols.io to enhance the reproducibility of your results. Protocols.io assigns your protocol its own identifier (DOI) so that it can be cited independently in the future. For instructions see: [https://journals.plos.org/plosone/s/submission-guidelines#loc-laboratory-protocols](https://track.editorialmanager.com/CL0/https:%2F%2Fjournals.plos.org%2Fplosone%2Fs%2Fsubmission-guidelines%23loc-laboratory-protocols/1/010f0197d1293b22-156e7551-058a-4ca6-b1f1-70caeaab1739-000000/jczsJRf6u1c8VxVOTmnwv8YUdXuG0j90Jrwm0EGWe6I=218). Additionally, PLOS ONE offers an option for publishing peer-reviewed Lab Protocol articles, which describe protocols hosted on protocols.io. Read more information on sharing protocols at [https://plos.org/protocols?utm_medium=editorial-email&utm_source=authorletters&utm_campaign=protocols](https://track.editorialmanager.com/CL0/https:%2F%2Fplos.org%2Fprotocols%3Futm_medium=editorial-email%26utm_source=authorletters%26utm_campaign=protocols/1/010f0197d1293b22-156e7551-058a-4ca6-b1f1-70caeaab1739-000000/b--LpXu6t9SQY5CmO5MJVAIQ5Fm6l_nJFxG7MgjbGlQ=218).
We look forward to receiving your revised manuscript.

Kind regards,

Iskra Alexandra Nola

Academic Editor

PLOS ONE

**Authors’ response:** In addition to the rebuttal letter, we submitted the 'Revised Manuscript with Track Changes' and the 'Manuscript'. In addition to the corrections made and marked in the manuscript in accordance with the reviewers’ suggestions, we have also corrected typographical errors, improved the style, and shortened certain sections to enhance clarity and reduce excessive length. We have also corrected the axis labels in Figures 1 to 4 (e.g., Number of vaccinated individuals, Years, Months, Number of promotional campaigns).

Journal Requirements:

When submitting your revision, we need you to address these additional requirements.

1. Please ensure that your manuscript meets PLOS ONE's style requirements, including those for file naming. The PLOS ONE style templates can be found at

<https://journals.plos.org/plosone/s/file?id=wjVg/PLOSOne_formatting_sample_main_body.pdf> and

[https://journals.plos.org/plosone/s/file?id=ba62/PLOSOne_formatting_sample_title_authors_affiliations.pdf](https://track.editorialmanager.com/CL0/https:%2F%2Fjournals.plos.org%2Fplosone%2Fs%2Ffile%3Fid=ba62%2FPLOSOne_formatting_sample_title_authors_affiliations.pdf/1/010f0197d1293b22-156e7551-058a-4ca6-b1f1-70caeaab1739-000000/3KuuBRApiMaHPyxLUgzrQfdFu9dK80XIzYaLio8Pg1Q=218)

2. We note that your Data Availability Statement is currently as follows: [All relevant data are within the manuscript and its Supporting Information files]

Please confirm at this time whether or not your submission contains all raw data required to replicate the results of your study. Authors must share the “minimal data set” for their submission. PLOS defines the minimal data set to consist of the data required to replicate all study findings reported in the article, as well as related metadata and methods (<https://journals.plos.org/plosone/s/data-availability#loc-minimal-data-set-definition>).
For example, authors should submit the following data:

-The values behind the means, standard deviations and other measures reported;

-The values used to build graphs;

-The points extracted from images for analysis.

Authors do not need to submit their entire data set if only a portion of the data was used in the reported study.
If your submission does not contain these data, please either upload them as Supporting Information files or deposit them to a stable, public repository and provide us with the relevant URLs, DOIs, or accession numbers. For a list of recommended repositories, please see <https://journals.plos.org/plosone/s/recommended-repositories>.

**Authors’ response:** We have also prepared and submitted depersonalized data related to this paper.
If there are ethical or legal restrictions on sharing a de-identified data set, please explain them in detail (e.g., data contain potentially sensitive information, data are owned by a third-party organization, etc.) and who has imposed them (e.g., an ethics committee). Please also provide contact information for a data access committee, ethics committee, or other institutional body to which data requests may be sent. If data are owned by a third party, please indicate how others may request data access.

**Authors’ response:** There are no ethical restrictions for this depersonalized database.

3. We notice that your supplementary tables are included in the manuscript file. Please remove them and upload them with the file type 'Supporting Information'. Please ensure that each Supporting Information file has a legend listed in the manuscript after the references list.

**Authors’ response:** We removed S1 Table and S2 Table from the main text and moved their titles after the reference list, while both tables were submitted as Supporting Information.

4. We are unable to open your Supporting Information file [data.zip]. Please kindly revise as necessary and re-upload.

**Authors’ response:** We have re-submitted the file [data.zip], which we believe can now be opened.

5. Please include captions for your Supporting Information files at the end of your manuscript, and update any in-text citations to match accordingly. Please see our Supporting Information guidelines for more information: <http://journals.plos.org/plosone/s/supporting-information>.

**Authors’ response:** We have done as indicated here.

6. Please review your reference list to ensure that it is complete and correct. If you have cited papers that have been retracted, please include the rationale for doing so in the manuscript text, or remove these references and replace them with relevant current references. Any changes to the reference list should be mentioned in the rebuttal letter that accompanies your revised manuscript. If you need to cite a retracted article, indicate the article’s retracted status in the References list and also include a citation and full reference for the retraction notice.

**Authors’ response:** We have formatted the references in accordance with the instructions provided here and the journal's requirement.

**Reviewers' comments:**

Reviewer's Responses to Questions

**Comments to the Author**

1. Is the manuscript technically sound, and do the data support the conclusions?

The manuscript must describe a technically sound piece of scientific research with data that supports the conclusions. Experiments must have been conducted rigorously, with appropriate controls, replication, and sample sizes. The conclusions must be drawn appropriately based on the data presented.

Reviewer #1: Yes

Reviewer #2: Partly

2. Has the statistical analysis been performed appropriately and rigorously?

Reviewer #1: Yes

Reviewer #2: Yes

3. Have the authors made all data underlying the findings in their manuscript fully available?
The [PLOS Data policy](http://track.editorialmanager.com/CL0/http:%2F%2Fwww.plosone.org%2Fstatic%2Fpolicies.action%23sharing/1/010f0197d1293b22-156e7551-058a-4ca6-b1f1-70caeaab1739-000000/Pjcj6E5doxS8lLreJSQviIzF7PnExiQWLyUcJw9hSBk=218) requires authors to make all data underlying the findings described in their manuscript fully available without restriction, with rare exception (please refer to the Data Availability Statement in the manuscript PDF file). The data should be provided as part of the manuscript or its supporting information, or deposited to a public repository. For example, in addition to summary statistics, the data points behind means, medians and variance measures should be available. If there are restrictions on publicly sharing data—e.g. participant privacy or use of data from a third party—those must be specified.

Reviewer #1: Yes

Reviewer #2: Yes

4. Is the manuscript presented in an intelligible fashion and written in standard English?
PLOS ONE does not copyedit accepted manuscripts, so the language in submitted articles must be clear, correct, and unambiguous. Any typographical or grammatical errors should be corrected at revision, so please note any specific errors here.

Reviewer #1: Yes

Reviewer #2: Yes

5. Review Comments to the Author

Please use the space provided to explain your answers to the questions above. You may also include additional comments for the author, including concerns about dual publication, research ethics, or publication ethics. (Please upload your review as an attachment if it exceeds 20,000 characters)

Response to Reviewers

**Reviewer #1:**

Abstract

• The sentence “...showed moderate effects. Educational efforts had limited influence.” would benefit from numerical clarity (e.g., correlation coefficients or p-values) to support the claims.

**Authors’ response:** Thank you for these valuable suggestions. We have added the appropriate text to the Abstract of the paper.

• Replace “among adolescents” with “among adolescents aged 9–19 years” for consistency with the main text.

**Authors’ response:** Thank you for this suggestion. For the sake of consistency, we have replaced the categories you mentioned throughout the manuscript.

Introduction
• Clarify the sentence “...regardless of type Serbia ranks third…” by correcting the punctuation: insert a period or semicolon between “type” and “Serbia.”

**Authors’ response:** Thank you for the remark. We have corrected the punctuation by inserting a [period/semicolon] between 'type' and 'Serbia' for clarity.

• Provide a citation for the “Health Policy paper” that defines HPV immunization as a “quick buy.”

**Authors’ response:** Thank you for this suggestion. We have inserted the reference number in the appropriate section of the manuscript.

• The aim statement is too long and somewhat repetitive. It could be tightened to a single paragraph focusing on the core research questions.

**Authors’ response:** Thank you for this valuable suggestion. We have shortened the aim of the study in accordance with your recommendation.

• Use consistent terminology (e.g., "nanovalent HPV vaccine" vs. "nonavalent HPV vaccine").

**Authors’ response:** Thank you for your comment. We have reviewed the manuscript and ensured consistent use of the term “nonavalent HPV vaccine” throughout.

• Clearly define whether the campaign’s reach extended beyond Novi Sad.

**Authors’ response:** Thank you for this suggestion. It is possible that we did not emphasize clearly enough that this study covered only the territory of a single city in Serbia—Novi Sad, which is the second largest in the country. We will add that the study was conducted exclusively in the territory of Novi Sad in the Aims (Introduction), Methods, and Discussion sections.

Materials and Methods

• Clarify what “address databases” were used—school-registered parent contacts? Was consent implied or granted?

**Authors’ response:** Thank you for your questions. As suggested, we have included a more detailed explanation of this topic in the relevant part of the Materials and Methods section.

• Include the time frame of implementation for each HPS (e.g., when were TV campaigns launched?).

**Authors’ response:** Thank you for these suggestions. As before, we have added a more detailed explanation of this topic in the relevant part of the Materials and Methods section.

• Elaborate on the regression model's specifications—how was the time lag introduced and validated?

**Authors’ response**: Thank you for your valuable comment regarding the specifications of the regression model and the introduction of the time lag. To investigate the delayed effects of promotional activities on vaccination uptake, we conducted a multiple linear regression analysis using time-shifted predictor variables. Specifically, for each promotional activity (e.g., “Open Door” sessions, distribution of printed materials, educational measures), the variable represented the activity’s scope in the previous month, while the dependent variable was the number of vaccinated individuals in the current month. A time lag of one month was applied based on the assumption that the impact of promotional activities may not be immediate but can manifest with some delay, which was particularly evident in the years 2023 and 2024 (see Fig. 3). The one-month interval was chosen as it realistically reflects the time needed for individuals to respond to the campaigns and decide to vaccinate. Importantly, this choice of lag was statistically validated, as the regression model incorporating the one-month lag explained a significantly higher proportion of variance (R² = 0.409, F(6,65) = 7.495, p < 0.001) compared to a model without any time lag. We believe this approach appropriately captures the temporal dynamics between promotional efforts and vaccination uptake.

Should the reviewer consider it useful, we are willing to incorporate a concise description of this modeling approach and the justification for the chosen time lag into the relevant section of the manuscript.

• Explain rationale for selecting the Mann–Whitney U test over t-tests or nonparametric alternatives.

**Authors’ response**: Thank you for your comment. The Mann–Whitney U test was selected over parametric t-tests for several reasons. First, the distribution of monthly vaccination data was highly skewed, and the sample sizes per group were relatively small, which violates the normality assumption required for independent-samples t-tests. This was confirmed by the Shapiro–Wilk test, which indicated statistically significant deviations from normality in most groups. Given these violations, the Mann–Whitney U test—a robust nonparametric alternative—was deemed appropriate, as it does not assume normality or homogeneity of variances and is well-suited for comparing two independent groups with non-normally distributed data.

These clarifications have now been included in the manuscript.

• Include a brief description of assumptions checked before using ANOVA and regression.

**Authors’ response**: Thank you for your comment. For ANOVA, the assumptions of normality and homogeneity of variances were assessed using the Shapiro–Wilk and Levene’s tests, respectively. The results indicated that the data for boys and girls did not consistently meet the assumption of normality (p < .05), and Levene’s test showed unequal variances between groups. Despite these limitations, ANOVA was retained for exploratory comparisons between sexes, given the equal group sizes and the value of planned post hoc analysis. All results were interpreted with caution, and nonparametric alternatives were applied to support the findings where appropriate. For the regression models, diagnostic checks confirmed that key assumptions were met. The P–P plot of standardized residuals indicated that residuals were approximately normally distributed. Visual inspection of residuals supported the assumptions of linearity and homoscedasticity. All Variance Inflation Factor (VIF) values ranged from 1.13 to 2.30, indicating that multicollinearity was not a concern. We have acknowledged these methodological considerations in the revised manuscript and advised readers to interpret the results accordingly.
Results

• Table 2 lists all p-values as “.000”—replace with “< .001.”

**Authors’ response**: Thank you for this suggestion. In Table 2, we have replaced “.000” with “< .001.”

• For correlation analysis (Table 3), consider grouping HPS by type (social marketing vs. educational) for clarity.

**Authors’ response**: Thank you for this. We have revised the layout of the table in accordance with your recommendation.
• Some figure captions (e.g., Fig 2, Fig 3) should describe what the reader should take away from the figure (e.g., “note peaks coinciding with Open Door campaigns”).

**Authors’ response**: Thank you for this valuable suggestion. We have added an appropriate description to the legends of Figures 2 and 3.

Discussion
• The first paragraph should more clearly summarize key findings before elaborating on implications.

**Authors’ response:** We appreciate this valuable suggestion. Accordingly, we have added a new introductory paragraph at the start of the Discussion section.

• Avoid vague statements like “a substantial body of research”; specify examples or summarize meta-analysis findings concisely.

**Authors’ response:** Thank you very much. We have added appropriate text in the Discussion section related to this topic.

• Discuss the potential impact of confounding factors—e.g., were other health campaigns running concurrently?

**Authors’ response:** Thank you for this insightful suggestion. Taking the overall concept of the Discussion into account, we have added a “Limitations of the study” section, and addressed the issue you raised under the third listed limitation.

Conclusion
• Consider highlighting the novelty of the “Open Door” approach and its potential for scale-up in similar settings.

**Authors’ response:** Thank you for this helpful advice. We have reformulated the conclusion in accordance with your suggested addition.

• Add a final sentence on next steps or policy implications.

**Authors’ response:** Thank you for this valuable suggestion. In response, we have added a final sentence to the conclusion section outlining potential next steps and policy implications based on our findings.

**Reviewer #2:**

I am grateful for the opportunity to review this article. The following may assist in improving the manuscript further. Regarding the spread of HPV and the increase in its complications, research on it is essential.

**Authors’ response:** Thank you very much for your thoughtful review and encouraging comments. We appreciate your recognition of the importance of research on HPV spread and its associated complications. We believe that our study contributes valuable epidemiological insights and evidence on the impact of health promotion strategies on HPV vaccination uptake, which are essential for informing future public health interventions

1- The data collection tool should be explained in the text.

**Authors’ response:** Thank you for this helpful comment. We have revised the Methods section to clarify the data collection process. Since the process of HPV vaccination data collection has been described, we have added relevant sections in the Materials and Methods (subsection “Health-Promotion Strategies (HPS) Until December 2024”) that relate to other important components, primarily the Health Promotion Strategies: Media Activities, Website Updates and Social Media Activity, Distribution of Health Education and Promotion Materials, Open Door Activities, Health Worker Education, and Education of Youth and Parents.
2. Since parental consent is required for adolescent vaccination, it is best to include and explain parental demographic characteristics in the results and discussion.

**Authors’ response:** Thank you for your thoughtful comment. We agree that parental demographic characteristics can provide valuable context when analyzing vaccination uptake among adolescents. However, this aspect was beyond the scope of our study. In accordance with our study objectives, we requested only fully anonymized data from the electronic immunization registry maintained by the Pediatric Department of the Primary Health Care Center Novi Sad. The dataset included only date of birth, gender, and date of HPV vaccine administration. No parental information was collected, and this has been clarified in the Materials and Methods section, as well as in the fifth listed limitation of the study.

3. Were the programs listed only offered to parents of adolescents? Include in the text.
**Authors’ response:** Thank you for your insightful comment. We have added the appropriate text to the Discussion section of the paper.

4. Did all parents of eligible adolescents have access to cell phones, websites, podcasts, etc., and were they literate enough to use them? Include in the text.

**Authors’ response:** Thank you for raising this important point. We did not assess parents' access to digital communication channels (e.g., cell phones, websites, podcasts) or their level of digital literacy, as this was beyond the scope of our study and not included in the dataset. However, recognizing the potential limitations in access and digital skills among certain segments of the population, we deliberately implemented a multi-modal communication strategy. In addition to electronic channels, the campaign also utilized printed materials (leaflets, brochures, posters) and in-person educational activities to ensure broader reach and accessibility for all parents. This issue has now been included as the sixth limitation of the study.

5. Since vaccination rates have been increasing over the years of the study, it should be justified that the “open door” approach had the greatest impact and was not due to prior information and other methods. Specify how confounding variables were controlled.

**Authors’ response:** Thank you very much for this suggestion. By way of explanation, we can presume that although vaccination rates increased across all years of the study, the most pronounced acceleration coincided with the implementation of the “Open Door” approach. While causality cannot be definitively established due to the observational nature of the data, the strength and consistency of the effect, as demonstrated by the regression analysis, support the relevance of this initiative. To minimize potential confounding, other promotional activities presented in this paper were included in the regression model alongside the “Open Door” intervention. In this model, only the “Open Door” initiative emerged as a statistically significant predictor of vaccination uptake. This strengthens the assumption that the observed increase is not solely attributable to accumulated exposure or prior awareness efforts, but rather reflects the unique contribution of the “Open Door” intervention. This issue has now been included in the Discussion section of the paper.

6. PLOS authors have the option to publish the peer review history of their article ([what does this mean?](https://track.editorialmanager.com/CL0/https:%2F%2Fjournals.plos.org%2Fplosone%2Fs%2Feditorial-and-peer-review-process%23loc-peer-review-history/1/010f0197d1293b22-156e7551-058a-4ca6-b1f1-70caeaab1739-000000/ChNbEk8w3Lr7zSD9zX06x9AQRVmT6MfOm108ayM-tkw=218)). If published, this will include your full peer review and any attached files.

If you choose “no”, your identity will remain anonymous but your review may still be made public.
**Do you want your identity to be public for this peer review?** For information about this choice, including consent withdrawal, please see our [Privacy Policy](https://track.editorialmanager.com/CL0/https:%2F%2Fwww.plos.org%2Fprivacy-policy/1/010f0197d1293b22-156e7551-058a-4ca6-b1f1-70caeaab1739-000000/lz7aiuKWyLDX4LN8Pq4zxs-W3AvykmgL9uYL_w0UwR0=218).

Reviewer #1: No

Reviewer #2: **Yes:** Mahnaz Azari.PhD midwifery, Reproductive Health Promotion Research Center, Ardebil University of Medical Sciences, Ardebil, Iran.
